# Effect of heat treatments on superconducting properties and connectivity in K-doped BaFe_2_As_2_

**DOI:** 10.1038/s41598-021-82325-x

**Published:** 2021-02-04

**Authors:** Chiara Tarantini, Chongin Pak, Yi-Feng Su, Eric E. Hellstrom, David C. Larbalestier, Fumitake Kametani

**Affiliations:** 1 Applied Superconductivity Center, National High Magnetic Field Laboratory, Florida State University, Tallahassee, FL 32310 USA; 2 Present Address: Oak Ridge National Laboratory, Oak Ridge, USA

**Keywords:** Superconducting properties and materials, Superconducting properties and materials

## Abstract

Fe-based superconductors and in particular K-doped BaFe_2_As_2_ (K-Ba122) are materials of interest for possible future high-field applications. However the critical current density (*J*_*c*_) in polycrystalline Ba122 is still quite low and connectivity issues are suspected to be responsible. In this work we investigated the properties of high-purity, carefully processed, K-Ba122 samples synthesized with two separate heat treatments at various temperatures between 600 and 825 °C. We performed specific heat characterization and *T*_*c*_-distribution analysis up to 16 T and we compared them with magnetic *T*_*c*_ and *J*_*c*_ characterizations, and transmission-electron-microscopy (TEM) microstructures. We found no direct correlation between the magnetic *T*_*c*_ and *J*_*c*_, whereas the specific heat *T*_*c*_-distributions did provide valuable insights. In fact the best *J*_*c*_-performing sample, heat treated first at 750 °C and then at 600 °C, has the peak of the *T*_*c*_-distributions at the highest temperatures and the least field sensitivity, thus maximizing *H*_*c2*_. We also observed that the magnetic *T*_*c*_ onset was always significantly lower than the specific heat *T*_*c*_: although we partially ascribe the lower magnetization *T*_*c*_ to the small grain size (< *λ*, the penetration depth) of the K-Ba122 phase, this behaviour also implies the presence of some grain-boundary barriers to current flow. Comparing the *T*_*c*_-distribution with *J*_*c*_, our systematic synthesis study reveals that increasing the first heat treatment above 750 °C or the second one above 600 °C significantly compromises the connectivity and suppresses the vortex pinning properties. We conclude that high-purity precursors and clean processing are not yet enough to overcome all *J*_*c*_ limitations. However, our study suggests that a higher temperature *T*_*c*_-distribution, a larger *H*_*c2*_ and a better connectivity could be achieved by lowering the second heat treatment temperature below 600 °C thus enhancing, as a consequence, *J*_*c*_.

## Introduction

Superconductivity in Fe-based compounds was first discovered in 2006 by Kamihara et al*.*^1^ when the LaOFeP (1111) phase was reported to have a critical temperature, *T*_*c*_, below 5 K. Shortly thereafter, a *T*_*c*_ high enough to be of interest for applications was demonstrated^2^ and other Fe-based superconductors (FBS) belonging to different families were then discovered with *T*_*c*_ up to ~ 55 K^3–5^. From the applications point of view, the interest in FBS is related to their possibly more affordable fabrication cost in form of wires or tapes when compared to cuprate high temperature superconductors (HTS). In fact research and development is ongoing for the realization of FBS wires or tapes suitable for High Energy Physics accelerator magnets^6^.

K-doped BaFe_2_As_2_ (K-Ba122) is the first FBS compound to have developed critical current density *J*_*c*_ exceeding 10^4^ A/cm^2^ at 10 T in wires^7^. The most recent tape performance has exceeded 5 × 10^4^ A/cm^2^ ^8–11^ and test coils have been produced^12^. For applications, high upper critical field *H*_*c2*_ and low anisotropy are considered particularly good attributes. Except for the 1111 phase, all FBS materials have high *H*_*c2*_ with small measured *H*_*c2*_ anisotropy^13–17^. However, the Fe(Se,Te) (11) and 122 phases are also strongly Pauli-limited at low temperature^18^ implying that the *H*_*c2*_ anisotropy might not be related to the effective mass anisotropy *γ*_*m*_ as predicted by the Ginzburg–Landau theory for single-band superconductors [where ${\gamma }_{{H}_{c2}}$ = (*M/m*)^1/2^ = *γ*_*m*_^1/2^]^19^. This issue is likely relevant in the 11 phase, which, despite the small ${\gamma }_{{H}_{c2}}$, shows behaviors typical of layered-structure materials with significant anisotropy^20^, as is observed also in the 1111 compounds^21,22^. However, such anomalies have never been observed in the 122 compounds, suggesting that the *γ*_*m*_ is moderate (although likely larger than ${{\gamma }_{{H}_{c2}}}^{2}$) making 122 the most promising FBS for applications. Despite the recent advances, *J*_*c*_ in 122 polycrystals or wires is still not as high as in single crystals or films^23–25^ and it is much smaller than the depairing current density^26^. There are two schools of thought about this polycrystalline degradation. One takes it to be an intrinsic degradation due to an suppression of superfluid density and *J*_*c*_ at misoriented grain boundaries (GBs)^27–29^, while the other invokes various extrinsic factors like cracks or impurities segregated to the GBs^30,31^. Since there is evidence for both effects, the importance of careful synthesis and understanding of the processing conditions is becoming increasingly important^8,32–34^.

In a recent study we investigated the effect of the synthesis conditions, focusing on the purity of the precursors, as well as the oxygen and moisture levels to which the powders were exposed during preparation^34^. We found that employing a high performance glovebox and high purity starting materials was essential to eliminate current-blocking impurities, such as Ba/K oxides, and to prevent the formation of metallic FeAs phase at grain boundaries. In this paper we studied samples prepared in these high purity conditions to investigate the effects of different heat treatments on the properties of K-Ba122, focusing on heat-treatment temperatures, which are the parameters most likely to affect the phase homogeneity. We performed specific heat characterizations to estimate the overall *T*_*c*_-distributions of K-Ba122. The advantage of this technique is that it provides direct information about the bulk properties of the superconducting phase, without being affected by connectivity, porosity or other extrinsic factors. We also correlated these results to magnetization, whole-sample *J*_*c*_ performance derived from magnetization measurements and TEM-evaluated microstructures to find a possible synthesis route for further improvement.

## Results

### Samples and initial electromagnetic characterizations

For this work six K-Ba122 samples were prepared with identical high purity elemental precursors ball milled in a high performance glovebox (see “Methods” and Ref.^34^). The samples belong to two series: the first series underwent a 1st heat treatment (HT) under hot isostatic pressure (HIP) conditions at temperatures varying between 600 and 825 °C, followed by a second ball-milling for better mixing before a second, higher HIP HT at 600 °C. The second series was first heat treated at 750 °C followed by a 2nd heat treatment at temperatures varying from 600 to 750 °C (ball-milling and HIP conditions were identical to the first series). One sample belongs to both series: it is identified in the figures by yellow background, lines or symbol edges. All characterizations were performed after completion of both heat treatments. The quality of the samples was first assessed by magnetic characterizations such as temperature dependence of the magnetization, to determine the magnetic *T*_*c*_ onset (*T*_*c,mag*_), and hysteresis loops at 4.2 K, to evaluate the field dependence of *J*_*c*_. Sample IDs (based on the HTs), heat treatment schedules and results from magnetic characterizations are summarized in Table 1. We found that the magnetic transitions have similar sharpness^34^ and their onset, *T*_*c,mag*_, ranges from 33.8 and 35.6 K, lower than the ~ 38 K *T*_*c,mag*_ of single crystals^4^. *J*_*c*_ also varies notably by a factor ~ 3–5, without any direct correlation to *T*_*c*,__*mag*_.

**Table 1:** Sample heat treatment schedules, magnetic onsets and critical current densities at 4.2 K at both 0.5 and 10 T.

	Sample ID	1st HT temp (°C)	1st HT time (h)	2nd HT temp (°C)	2nd HT time (h)	*T*_*c,mag*_ (K)	*J*_*c*_(0.5 T,4.2 K) (A/cm^2^)	*J*_*c*_(10 T,4.2 K) (A/cm^2^)
Varying 1st HT temperature	600/600	600	20	600	10	33.8	1.07 × 10^5^	1.08 × 10^4^
	675/600	675				34.5	1.40 × 10^5^	1.25 × 10^4^
	**750/600**	**750**				**34.4**	**1.84 × 10**^**5**^	**1.61 × 10**^**4**^
	825/600	825				34.3	1.16 × 10^5^	1.15 × 10^4^
Varying 2nd HT temperature	**750/600**	750	20	**600**	10	**34.4**	**1.84 × 10**^**5**^	**1.61 × 10**^**4**^
	750/675			675		35.6	0.82 × 10^5^	0.59 × 10^4^
	750/750			750		35.2	0.61 × 10^5^	0.33 × 10^4^

In the two series the same sample is marked with bold fonts.

### Specific heat characterization, *T*_*c*_-distribution analysis and comparison with other properties

**Figure 1:**
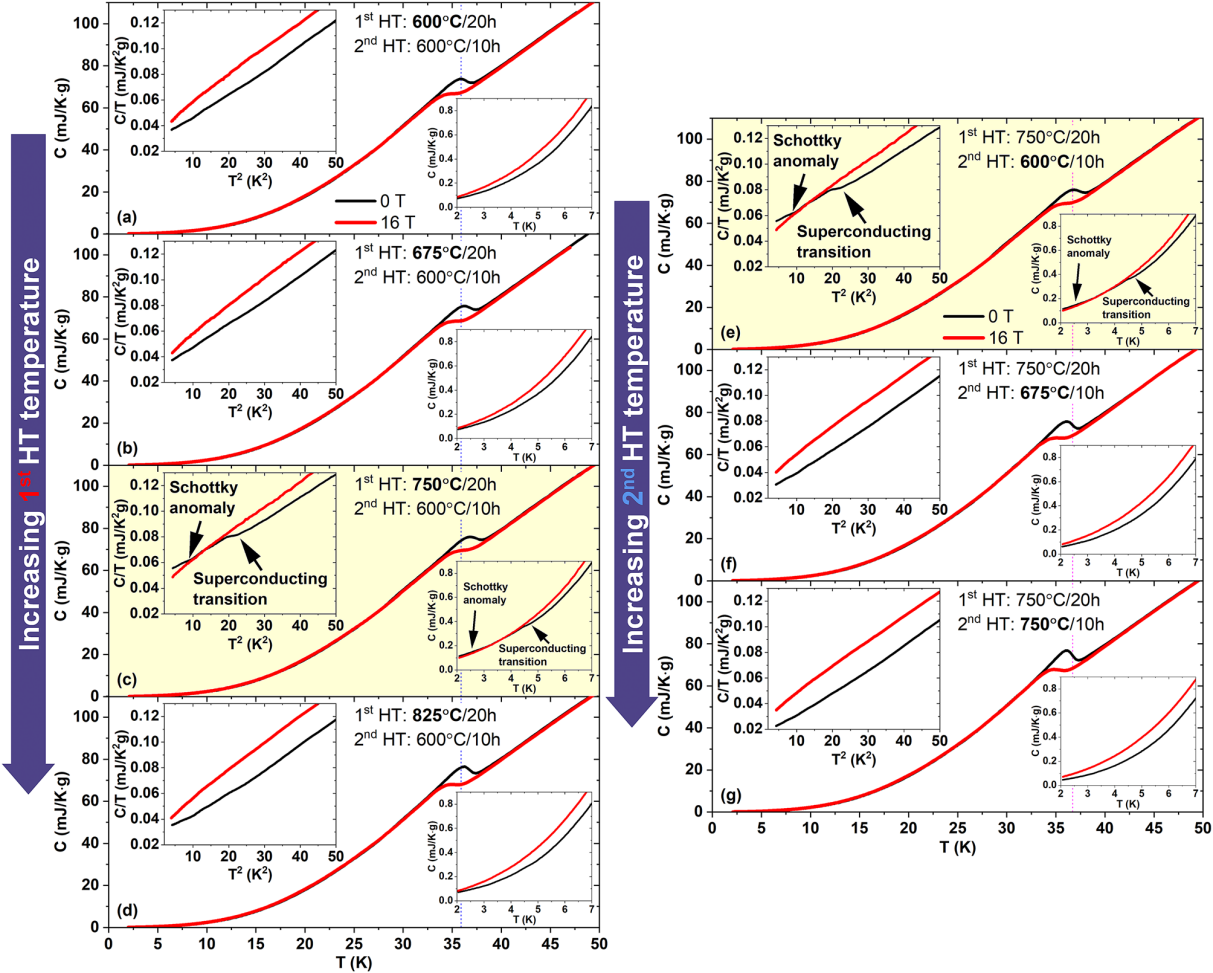
Specific heat characterization at 0 and 16 T for six K-Ba122 samples after different heat treatments. (**a**–**d**) the temperature dependence for the samples synthetized by varying the 1st heat treatment temperature between 600 and 825 °C and the 2nd at 600 °C. (**e**,**f**) similar data for the samples heat treated at 750 °C with the 2nd heat treatment varied between 600 and 750 °C. In the insets, the low temperature magnification of the C versus T and/or C/T versus T^2^ curves.

The specific heat measurements performed at 0 and 16 T on the six samples are reported in Fig. 1. In all cases, we observed superconducting transitions of the K-Ba122 phase at ~ 35–38 K with a very little shift at 16 T, as expected from the high *H*_*c2*_. Most samples appear rather homogeneous with the exception of the sample heat treated at 750 °C/20 h + 600 °C/10 h (sample ID 750/600) belonging to both series and marked in yellow. In this case, we identify a secondary superconducting transition at low temperatures of about 4–5 K [probably due to KFe_2_As_2_, see insets, in particular the C/T versus T^2^ plot in Fig. 1c,e] as well a clear sign of a Schottky-like anomaly. This can be recognized by the anomalous crossover at low temperature (~ 3 K) with the specific heat at 0 T larger than at 16 T, which is opposite of what occurs in a standard superconductor. Although not as obvious, the Schottky-like anomaly appears to affect all samples as suggested by the mild curvatures at low temperature.

In Fig. 1a–d we can see the effect of varying the 1st HT temperature at constant 600 °C for the second HT. At 0 T the main transition is obviously sharper at 825 °C, but neither the broadening nor the transition position monotonically change with HT temperature. In Fig. 1e–g we observe the effect of variable 2nd HT temperature (after the first HT at 750 °C). In this case the 0 T transition becomes sharper with increasing the 2nd HT temperature but it also shifts to lower temperature. In both series, the general trend becomes hard to follow when 16 T is applied because of the typical high-field broadening.

**Figure 2:**
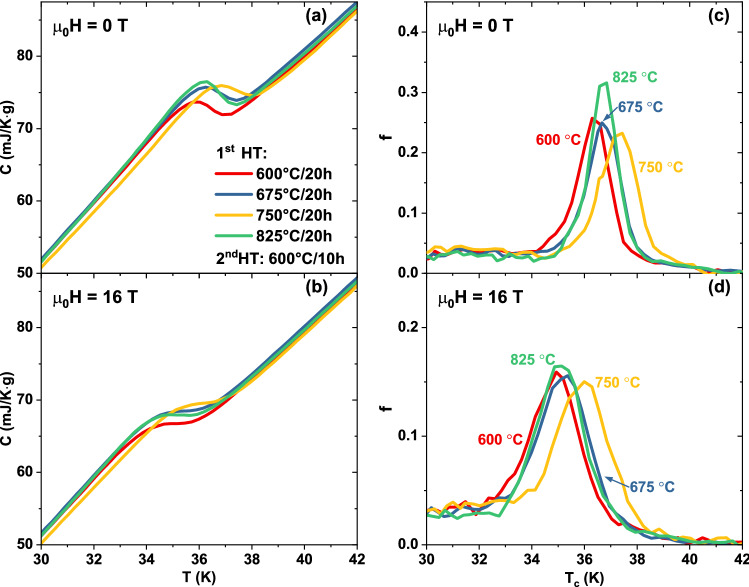
Specific heat transitions and *T*_*c*_-distribution of the K-Ba122 samples prepared varying the 1st HT temperature. (**a**,**b**) magnification of the main superconducting transitions at 0 and 16 T. (**c**,**d**) correspondent *T*_*c*_-distributions calculated as explained in the text.

**Figure 3:**
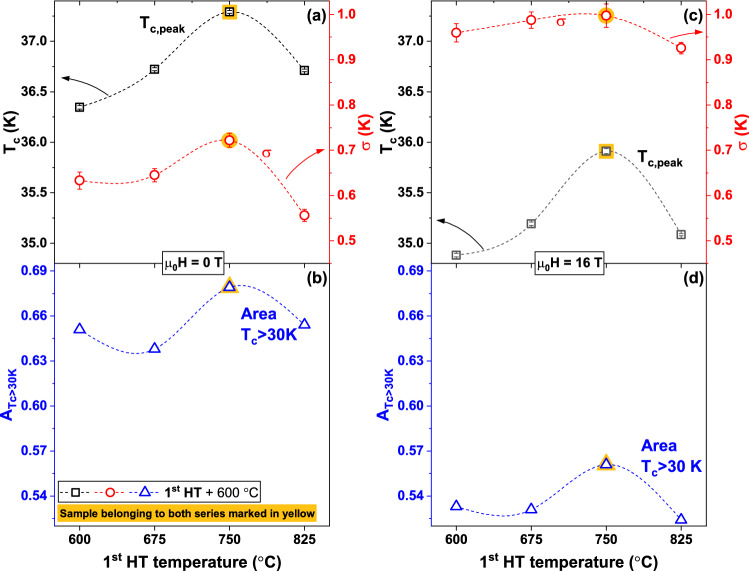
*T*_*c*_-distribution properties of the K-Ba122 samples as a function of the 1st HT temperature. (**a**) position and width of the main peaks in the *T*_*c*_-distributions at 0 T obtained in Fig. 2. (**b**) area of the 0 T *T*_*c*_-distribution above 30 K. (**c**,**d**) similar data for the 16 T results.

To better quantify the changes caused by the different heat treatments, we analysed the specific heat data with the *T*_*c*_-distribution model first developed for A15 compounds^35^ and employed to systematically investigate small differences in Nb_3_Sn produced by heat treatment^36,37^ or doping^38,39^. This model was also previously employed to investigate FBS materials^40,41^. Figure 2 shows the *T*_*c*_-distribution, *f*(*T*_*c*_) (where ${\int }_{0}^{{T}_{c,Max}}f\left({T}_{c}\right)\mathrm{d}{T}_{c}=1$), obtained varying the 1st HT temperature (together with the magnifications of the transitions in Fig. 2a,b) and Fig. 3 summarizes the results in terms of the main peak properties, *T*_*c,peak*_ and σ (defined by the peak centre and width of a Gaussian fit over the linearized background), and the area of the *T*_*c*_-distribution above 30 K, *A*_*Tc*>*30 K*_. The 30 K threshold was chosen to include the main peaks in all samples at both 0 and 16 T as a measure of the high-*T*_*c*_ portion of the distribution; this allows a sample-to-sample comparison in a temperature range that most likely affects to the overall performance. *f*(*T*_*c*_) at 0 T (Fig. 2c) reveals that *T*_*c,peak*_ increases by about 1 K from ~ 36.3 to ~ 37.3 K (Fig. 3a) on increasing the 1st HT temperature from 600 °C (sample 600/600) to 750 °C (sample 750/600). At the same time, σ slightly increases (from 0.63 to 0.72 K). For the 1st HT at 825 °C *T*_*c*,__*peak*_ drops to ~ 36.7 K but with a clear sharpening of the peak (σ ~ 0.56 K). *A*_*Tc*>*30 K*_ (Fig. 3b), varying from 0.64 to 0.68, does not follow the same exact trend but the maximum is still reached at 750 °C. At 16 T (Fig. 3c,d) the overall trends are the same with the obvious differences that *T*_*c*,__*peak*_ and *A*_*Tc*>*30 K*_ are lower than at 0 T, whereas the peak width σ increases; however, the peak broadening is quite similar in all samples (σ ~ 0.93–1.00 K). Figure 2d also shows that, at 16 T, the *T*_*c*_-distribution of sample 750/600 is more clearly separated from those of the other samples.

**Figure 4:**
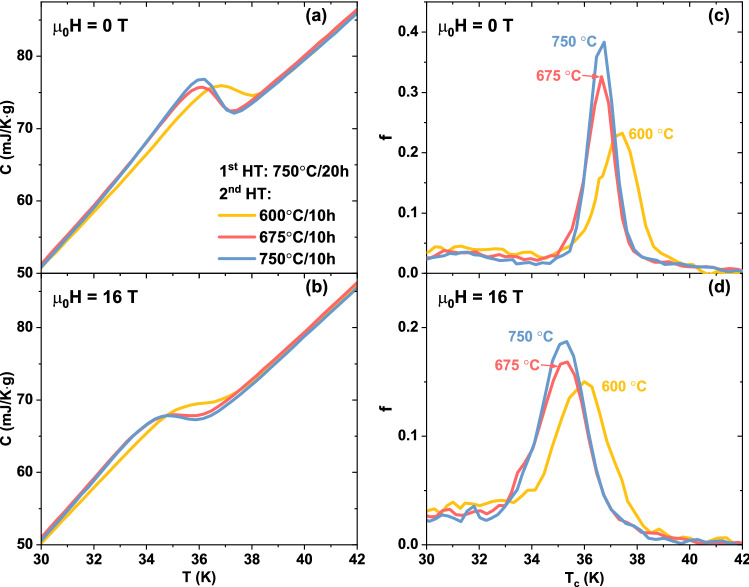
Specific heat transitions and *T*_*c*_-distribution of the K-Ba122 samples prepared varying the 2nd HT temperature. (**a**,**b**) magnification of the main superconducting transitions at 0 and 16 T. (**c**,**d**) correspondent *T*_*c*_-distributions calculated as explained in the text.

**Figure 5:**
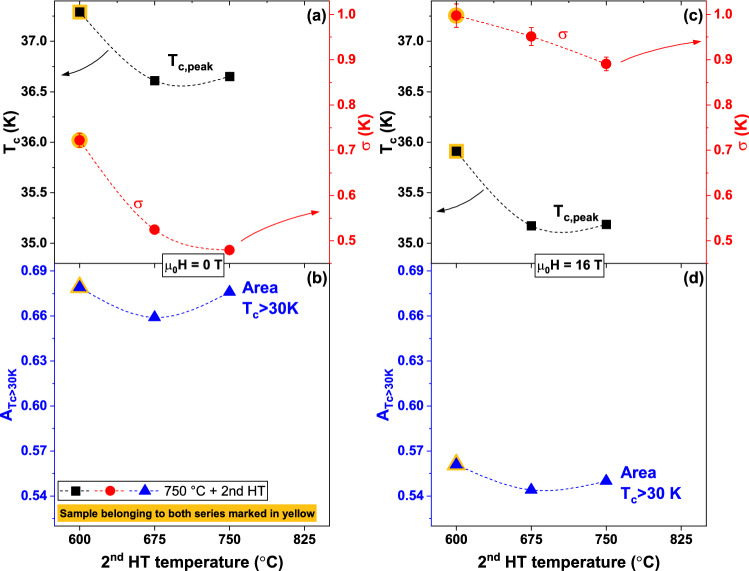
*T*_*c*_-distribution properties of the K-Ba122 samples as a function of the 2nd HT temperature. (**a**) position and width of the main peaks in the *T*_*c*_-distributions at 0 T obtained in Fig. 2. (**b**) area of the 0 T *T*_*c*_-distribution above 30 K. (**c**,**d**) similar data for the 16 T results.

A similar analysis was performed to investigate the effect of the 2nd HT and the results are reported in Figs. 4 and 5 (magnifications of the transitions in Fig. 4a,b). Starting from 600 °C (sample 750/600, which belongs to both series) *T*_*c*,__*peak*_ at 0 T drops by about 0.7 K with increasing the 2nd HT temperature (Fig. 5a) but simultaneously the transition becomes much sharper with σ going from 0.72 to 0.48 K. *A*_*Tc*>*30 K*_ does not have a monotonic behaviour (Fig. 5b) and it varies from a maximum of 0.68 at 600 °C to a minimum of 0.66 at 675 °C. As before, the 16 T data (Figs. 4c,d, 5c,d) follow the same general trend again with only small σ difference between samples.

**Figure 6:**
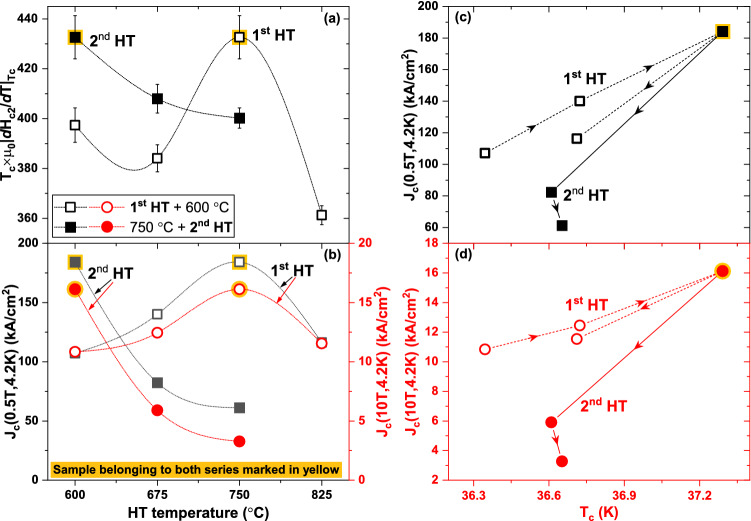
Effect of the heat treatment on the electromagnetic properties of K-Ba122. (**a**) ${T}_{c}\times {\left.d{H}_{c2}/dT\right|}_{{T}_{c}}$ and (**b**) low and high-field *J*_*c*_ at 4.2 K as a function of the heat treatment temperature. (**c**) low and (**d**) high-field *J*_*c*_ at 4.2 K as a function of the peak position of the *T*_*c*_-distribution. The arrows indicate the increasing HT temperature.

A direct comparison of the specific heat analysis of all samples shows that the 750/600 HT produces the least field-sensitive K-Ba122 transition. In fact, applying 16 T to this sample, the peak shifts by only 1.38 K, whereas in the other samples it ranges from 1.44 to 1.62 K (Figs. 2c,d and 4c,d). The suppressed field-sensitivity of this sample is also evident by the change of σ going from 0 to 16 T: in fact σ increases by less than 39% in 750/600, by 51–67% in the other samples belonging to the 1st series (Fig. 3a,c) and as high as 81–86% in the other samples belonging to the 2nd series (Fig. 5a,c). Because of the complex multiband, Pauli-limited and possibly FFLO-affected nature of these materials (FFLO: Fulde-Ferrell-Larkin–Ovchinnikov)^42–44^, the WHH (Werthamer-Helfard-Hohenberg) model^45^ cannot actually be used to estimate *H*_*c2*_(0) and very high field measurements would be necessary for fitting with models suitable for FBS^15,18^. However, since the low-temperature performance is expected to be affected by both *T*_*c*_ and the *H*_*c2*_ slope near *T*_*c*_, we can use ${T}_{c}\times {\left.d{H}_{c2}/dT\right|}_{{T}_{c}}$ (calculated from the 0–16 T *T*_*c*,__*peak*_) as a measure of the low temperature performance (n.b. ${T}_{c}\times {\left.d{H}_{c2}/dT\right|}_{{T}_{c}}$ does not provide an estimation of *H*_*c2*_). The ${T}_{c}\times {\left.|d{H}_{c2}/dT\right|}_{{T}_{c}}$ trend in Fig. 6a is clearly different from those in Figs. 3a and 5a. For instance, samples 675/600 and 825/600 (belonging to the 1st series) have the two lowest ${T}_{c}\times {|\left.d{H}_{c2}/dT\right|}_{{T}_{c}}$ values despite having the 2nd and 3rd best *T*_*c*,__peak_. Having both the highest *T*_*c*,__*peak*_ and the highest *H*_*c2*_ slope, this estimation suggests that sample 750/600 could have a low-temperature *H*_*c2*_ from 6 to 20% higher than any other samples.

In order to correlate the effects of heat treatments and *T*_*c*_-distributions to the *J*_*c*_ performance, we plot *J*_*c*_(4.2 K) at low and high fields (0.5 and 10 T) as a function of the HT temperature (Fig. 6b) and as a function of the peak *T*_*c*_ (Fig. 6c,d, discussed more broadly in the discussion). In both series the trend is in general similar to that observed for *T*_*c*_ as a function of HT temperature (compare Figs. 3a and 5a) with the best performing sample being 750/600, the one with the highest *T*_*c,peak*_. However, Fig. 6c,d show that increasing the 1st HT temperature up to 825 °C (sample 825/600) and increasing the 2nd HT temperature above 600 °C (samples 750/675 and 750/750) appears to have detrimental effect on *J*_*c*_ regardless of *T*_*c*_.

### Nanostructural characterization and compositional analysis

**Figure 7:**
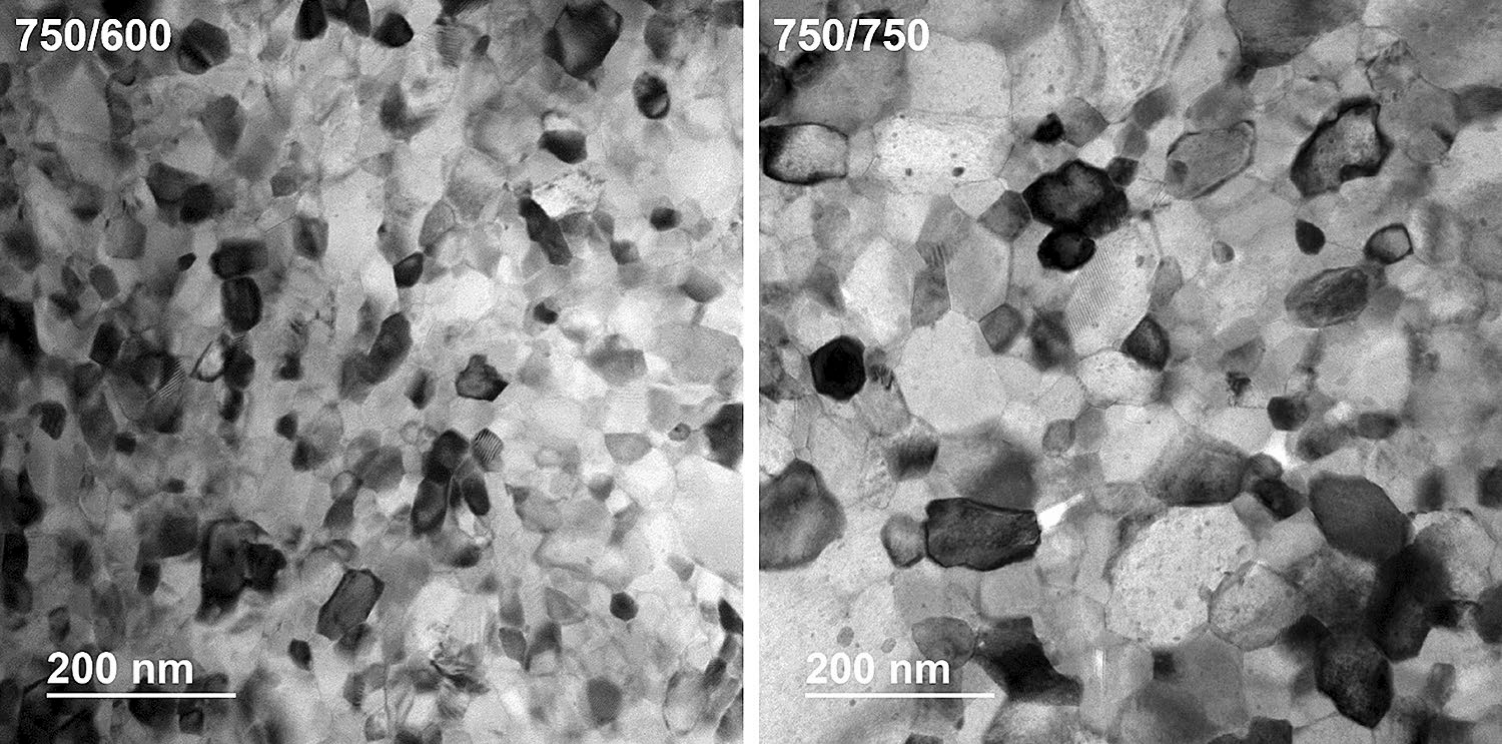
TEM images of K-Ba122 samples. Representative grain structures of two samples heat treated at 750 °C/20 h + 600 °C/10 h and 750 °C/20 h + 750 °C/10 h showing a clear difference in grain size.

Transmission electron microscopy (TEM) was performed on several samples. Figure 7 shows two representative samples with very different properties: the best performing sample, 750/600, and the lowest *J*_*c*_ sample with the highest 2nd HT temperature, 750/750. They show rather clean grain structures obtained thanks to the high purity precursors and the careful clean processing^34^, without the K, FeAs and BaO segregations observed in less pure samples^34,46^. In order to understand the *T*_*c*_ difference estimated by magnetization and specific heat, we evaluated the average grain size for the samples in Fig. 7. We found a clear difference in grain size in these two samples and we estimated the average values as 37 and 77 nm for 750/600 and 750/750, respectively. It is interesting to notice that in both cases the grain sizes are rather small when compared to samples prepared by other processing techniques (see for instance refs.^31,47^ with average grain size > 300 nm^47^).

Energy dispersive X-ray spectroscopy (EDS) was performed on the samples belonging to the 2nd series. The average compositions were obtained by performing EDS on about 40 K-Ba122 grains. The overall chemical compositions were obtained normalizing to (Ba + K) = 1 and we found the following: Ba_0.61_K_0.39_Fe_1.98_As_2.34_ for 750/600, Ba_0.64_K_0.36_Fe_2.42_As_2.26_ for 750/675 and Ba_0.63_K_0.37_Fe_2.47_As_2.27_ for 750/750. The best performing sample, 750/600, has an optimal Ba/K content explaining its highest *T*_*c*,*peak*_, whereas the other two samples are slight K-poor probably causing their suppressed *T*_*c*,*peak*_. All three samples are slightly As-rich but the effect of such off-composition is presently unclear.

## Discussion

The *T*_*c*_-distribution of K-Ba122 clearly reveals that the sample with the 1st at 750 °C and the 2nd HT at 600 °C has the highest-*T*_*c*_ peak, despite also having the largest peak broadening. Moreover, this sample is also the least field-dependent with a small in-field *T*_*c*,__*peak*_ shift, leading to its highest *H*_*c2*_, even with a marked Schottky-like anomaly at low temperature. In general, the Schottky anomaly is caused by lifting the degeneracy of the states of the paramagnetic spins in magnetic impurities and it was also observed in K-Ba122 single crystals^48^ (KFe_2_As_2_ also showed a Schottky anomaly but at a very low temperature, below 0.2 K^49^, outside the range of our measurements). This consideration and the fact this anomaly is less obvious in samples with a lower-*T*_*c*_ distribution suggest that the Schottky-like feature might be an intrinsic characteristic of the highest-*T*_*c*_ (or more K-rich) K-Ba122 phase, rather than an extrinsic residual secondary phase effect. What is clear is that Schottky-like anomaly does not appear to have negative impact on the K-Ba122 superconducting properties (*T*_*c*_, *H*_*c2*_ and *J*_*c*_).

Another important observation is that, whereas the 1st HT requires an intermediate optimal temperature, increasing the 2nd HT temperature above 600 °C causes a clear suppression of *T*_*c*,__*peak*_ and *H*_*c2*_ (Figs. 5a and 6a), despite the sharpening of the transitions. This suggests that lowering the 2nd HT temperature below 600 °C could be beneficial to further improve the bulk properties of the superconducting phase. However, this will have to be carefully investigated to verify what the effect on the connectivity is.

Comparing the specific heat characterizations with the other electromagnetic superconducting properties, we first notice the surprisingly large difference with the magnetization onsets, with *T*_*c*,__*mag*_ (Table 1) being much lower than both the specific heat onset and *T*_*c*,__*peak*_ (magnetization and specific heat onsets are almost identical in single crystals^48^). In all samples *T*_*c*,__*mag*_ falls below the main peaks of the *T*_*c*_-distributions, with about 50% of the superconducting volume fraction being above *T*_*c*,__*mag*_. For comparison, in similarly analyzed Nb_3_Sn, the magnetization onset was observed with about 10.5% of the phase in the superconducting state and we estimated that a percolative path around the core through the not well-connected Nb_3_Sn large grains was established at about 17.5%^37^. This means that an unexpectedly large superconducting volume fraction (~ 50%) is needed in K-Ba122 to produce any detectable magnetic shielding. As shown by the TEM characterization, the majority of the sample heat treated at 750/600 consists of small grains with an average diameter of ~ 37 nm. This size is much smaller than the penetration depth in K-Ba122, where *λ*_*ab*_ at low temperature is about 200–250 nm^50,51^. This means that single grains would generate very little shielding even at low temperature, i.e. a tiny magnetization signal, and approaching *T*_*c*_ this signal would be even smaller, because *λ* diverges. Although the small grain size might partially explain the large difference between specific heat and magnetization transitions, clusters of superconducting grains should generate a detectable signal with much less than 50% of the superconducting volume fraction (a complete 3D percolation path in bulk samples should be obtained with ~ 20% of the grains in the superconducting state^52^). Other possible reasons for this large difference are (1) connectivity issues and/or (2) the K-Ba122 anisotropy. (1) Extrinsic connectivity issues could be caused by secondary phases, which however are reduced to a minimum by the high purity synthesis process and are detected by specific heat measurements only in the best sample. The presence of micro-cracks or porosity affecting the connectivity cannot be excluded and it was, indeed, deduced by the TEM characterization in Ref.^34^ where a decrease in density at GBs was observed. Systematic studies changing for instance the cooling rate or lowering the 2nd HT temperature, as suggested above, should be considered to verify the possible effect of a reduced thermal stress. Since this clearly is a multi-parameter problem, HT time should be investigated as well in order to optimize connectivity and grain size. (2) As for the anisotropy, although an advantage of K-Ba122 is frequently considered its small anisotropy, this refers to its *H*_*c2*_ anisotropy, which decreases from ~ 2.5–3.5 at *T*_*c*_ toward 1 at low temperature^15,16^. However, as mentioned above, ${\gamma }_{{H}_{c2}}$ is affected by the Pauli limit, whereas ${\gamma }_{\xi}$, which depends on the mass anisotropy, does not and it might differ from ${\gamma }_{{H}_{c2}}$ (in contrast to *λ*, *ξ* cannot be directly measured). Since the depairing current density, *J*_*d*_, is inversely proportional to *λ*^2^*ξ*, in the low field limit we can assume that the in plane and out of plane *J*_*c*_ can be expressed by ${J}_{c}^{H//c}\sim 1/{{\lambda }_{ab}}^{2}{\xi }_{ab}$ and ${J}_{c}^{H//ab}\sim 1/{\lambda }_{ab}{\lambda }_{c}\sqrt{{\xi }_{ab}{\xi }_{c}}$ (taking into account the effective *λ* and *ξ* parameters). Their ratio then becomes ${J}_{c}^{H//c}/{J}_{c}^{H//ab}\sim {\lambda }_{c}/{\lambda }_{ab}\cdot \sqrt{{\xi }_{c}/{\xi }_{ab}}={\gamma }_{\lambda }/\sqrt{{\gamma }_{\xi }}$,where ${\gamma }_{\lambda }$ is ~ 7–7.3 at 0 K and ~ 2.5 near *T*_*c*_^51^. If ${\gamma }_{\xi }= {\gamma }_{{H}_{c2}}$, ${J}_{c}^{H//c}/{J}_{c}^{H//ab}$ would range between ~ 7–7.3 at 0 K to ~ 1.6–1.9 at *T*_*c*_. Considering the opposite limiting case with ${\gamma }_{\xi }= {\gamma }_{\lambda }$, ${J}_{c}^{H//c}/{J}_{c}^{H//ab}$ would range from ~ 2.6–2.7 at 0 K to ~ 1.6–1.9 at *T*_*c*_; however, this is unlikely because, as mentioned in the Introduction, a ${\gamma }_{\xi }$ as large as 7 would cause behaviors typical of layered-structures, which have never been observed in the 122 phase. In both limiting cases, the *J*_*c*_ anisotropy near *T*_*c*_ and at small applied field is low, so it should not cause impediments to the creation of roughly uniform percolative paths, leaving extrinsic factors as a likely contributor to the onset difference between magnetization and specific heat. It is interesting to note that, in the ${\gamma }_{\xi }= {\gamma }_{{H}_{c2}}$ case, a low-temperature *J*_*c*_ anisotropy above 7 would likely be quite detrimental for the current flow even in well-connected samples, causing more significant deviation of the current percolative paths.

A comparison between the specific heat results and *J*_*c*_ behavior provides additional information regarding the effect of the heat treatment on the connectivity and pinning properties. The low-field *J*_*c*_ performance should be mostly determined by *T*_*c*_. Figure 6c shows that, varying the 1st HT, *J*_*c*_ linearly increases with *T*_*c*_ up to the 1st HT temperature of 750 °C. The sample with the 1st HT at 825 °C (with about the same *T*_*c*_ as the sample heat-treated at 675 °C) clearly lies below this trend line with a 17% lower *J*_*c*_. This indicates that increasing the 1st HT temperature to 825 °C causes a slight decrease in connectivity. Increasing the 2nd HT temperature has an even greater effect on the low-field *J*_*c*_, making it 37 to 54% lower than for the best behaving trend followed by the 1st series and indicating that the connectivity is severely compromised. At high-fields *J*_*c*_ is supposed to be affected by additional parameters, such as *H*_*c2*_ and the pinning efficiency, whose contributions are in general hard to separate. However, the *J*_*c*_(10 T) versus *T*_*c*_ plot (Fig. 6d) for the 1st sample series has roughly the same trend observed at 0.5 T suggesting that those additional parameters are not playing a significant role. In each sample belonging to the 1st series *J*_*c*_(10 T) drops to ~ 9–10% of respective 0.5 T values, suggesting similar pinning performance in these four samples. For the 2nd sample series, increasing the HT temperature above 600 °C causes a more marked in-field suppression, with *J*_*c*_(10 T) being only ~ 5–7% of the 0.5 T values, likely due to a combination of reduced *H*_*c2*_ and pinning efficiency. Since grain boundaries are assumed to be the main pinning centres, the reduced pinning efficiency can be caused by the increase in grain size (decrease of GB density) observed by TEM (Fig. 7). The pinning efficiency can also be suppressed by the annealing of intragrain defects (such as point defects, which cannot be observed by TEM) due to the high temperature synthesis.

## Conclusions

In this work, we performed a systematic investigation of the effects of heat treatment on the superconducting properties of K-Ba122, by first varying the 1st HT temperature between 600 and 825 °C (keeping the 2nd at 600 °C) and then by changing the 2nd HT temperature from 600 to 750 °C (keeping the 1st at 750 °C). We found that the peak position of the 0 T *T*_*c*_-distribution is shifted at the highest temperature after performing the 1st and 2nd heat treatments at 750 and 600 °C, respectively. Despite a marked sign of Schottky-like anomaly at low temperature, this sample also shows the weakest field dependence, an advantage for the low-temperature performance. We observed a clear difference between the magnetization and specific heat transition which is likely being caused by a combination of the small grain size and extrinsic connectivity effects. In fact, because the grains are much smaller than the penetration depth, a large fraction of the sample may need to be in the superconducting state to allow current percolation and so shielding of the sample. However, since we estimated this fraction in about 50%, much larger than both the Nb_3_Sn estimated value and the theoretical percolation threshold, extrinsic factors, like micro-cracks, affecting the connectivity are likely to play a role. To better understand the particular effect of the heat treatment on the connectivity and pinning properties, we compared the low and high field *J*_*c*_ to the peak position of the *T*_*c*_-distribution. We found that changing the 1st HT temperature up to 750 °C has little effect on the connectivity and the low-field *J*_*c*_ varies linearly with *T*_*c*_. On the other hand, a further increase in the 1st HT temperature or increasing the 2nd HT temperature is clearly detrimental for the connectivity. By varying the 1st HT, *J*_*c*_(10 T) versus *T*_*c*,*peak*_ shows a similar trend to that found at low-field suggesting no significant changes in the pinning efficiency in these four samples. On the contrary *J*_*c*_(10 T) of the samples that underwent a higher-temperature 2nd HT are more strongly affected indicating a reduction of the pinning efficiency as well, possibly caused by grain growth or annealing of intragrain defects. These results indicate a possible synthesis route for improvement. In fact, since the best bulk properties (highest *T*_*c,peak*_ and highest *H*_*c2*_), and the best connectivity and *J*_*c*_ are found in the sample heat treated at the lowest tested 2nd HT temperature, we think that lowering the temperature even further, or decreasing the cooling rate, could be valuable both to improve the phase properties and to eliminate possible micro-cracks caused by the thermal stress.

## Methods

### Synthesis of K-doped BaFe_2_As_2_

Polycrystalline Ba_0.6_K_0.4_Fe_2_As_2_ bulk samples were synthesized using high purity elemental materials (≥ 99.9% for Ba, ≥ 99.95% for K, ≥ 99.99% for Fe and ≥ 99.9999% for As) with a nominal initial composition of Ba_0.6_K_0.44_Fe_2_As_2_ to compensate for the possible loss of K during the synthesis. The powders were mixed in a high-performance glove box (O_2_ ≤ 0.005 ppm and H_2_O ≤ 0.06 ppm), ball-milled for 6 h, sealed first in a Nb crucible and then in a steel one. The crucible was then compressed by cold isostatic pressing at 276 MPa and then heat treated by hot isostatic pressing (HIP) at 9.7 MPa for 20 h (1st HT); the samples were heated up in 12 h, to prevent abrupt reaction of the powders, and cooled down at 6 °C/min. Then samples were then grounded and ball-milled for 1 h, sealed and compressed similarly to the first processing. The second HIP heat treatment was performed at 193 MPa for 10 h (2nd HT); since the K-Ba122 phase is already formed at this stage, the samples were heated up in 1 h. The cooling rate was 4 °C/min. The heating information for the samples are summarized in Table 1 and more details of equipment and processing can be found in Ref.^34^.

### Specific heat and magnetic characterizations

Specific heat measurements were performed on samples of about 11 mg at 0 and 16 T in a 16 T Quantum Design physical property measurement system (PPMS) and analysed with Wang’s model^39^ with n = 4.5. This is a phenomenological model with the limitations discussed in Refs.^37,39^. The superconducting transitions were evaluated by magnetic measurements in a Quantum Design SQUID magnetometer MPMS XL5. The *H*_*c2*_ slope was estimated by the peak positions of the *T*_*c*_-distributions, *T*_*c*,__*peak*_, at 0 and 16 T in order to calculate ${T}_{c}\times {\left.d{H}_{c2}/dT\right|}_{{T}_{c}}$. The temperature dependence of the magnetization was measured after zero-field cooling with an applied field of 2 mT. Hysteresis loops were performed at 4.2 K in a Oxford Instruments 14 T Vibrating Sample Magnetometer (VSM) on samples with 3 × 1 × 0.3 mm^3^ size and the magnetization *J*_*c*_ was calculated using the Bean model^53,54^.

### Nanostructural characterization and compositional analysis

TEM imaging were performed in a JEOL JEM-ARM200CF Cs-corrected cold field emission scanning transmission electron microscope equipped with Oxford X-MaxN 100TLE SDD energy dispersive X-ray spectroscopy (EDS) detector. The TEM specimens were made either by mechanical polishing followed by ion milling in Gatan PIPS, or by using Focused Ion Beam in a Thermo Fisher Scientific/FEI Helios G4 UC Dual Beam Focused Ion Beam/Field Emission Scanning Electron Microscope. The average grain sizes were estimated by line-intersection method characterizing 15–20 different areas on each sample.
